# The Current and Future Role of MRI and PSMA-PET/CT in Diagnosing Oligometastatic Prostate Cancer

**DOI:** 10.1097/RLI.0000000000001264

**Published:** 2026-01-14

**Authors:** Tom W.J. Scheenen, Ansje S. Fortuin, Daniela E. Oprea-Lager, Maarten de Rooij

**Affiliations:** Department of Medical Imaging, Radboud University Medical Center, Nijmegen, The Netherlands (T.W.J.S., A.S.F., D.E.O.L., M.D.R.); Erwin L. Hahn Institute for Magnetic Resonance Imaging, Essen, Germany (T.W.J.S.); Department of Radiology, Ziekenhuis Gelderse Vallei, Ede, The Netherlands (A.S.F.)

**Keywords:** prostate cancer, USPIO, PSMA, whole-body MRI, lymph nodes, metastases, prostate-specific membrane antigenultrasmall superparamagnetic iron oxide

## Abstract

This review describes the role of different imaging techniques in the initial diagnosis and staging of prostate cancer (PCa), with a focus on oligometastatic disease. Men with an elevated prostate-specific antigen level and/or with an abnormal digital rectal exam are subject to a multiparametric MRI examination to identify a possible lesion in the prostate. In patients with high-risk or intermediate-risk disease with an unfavorable prognosis, additional imaging with prostate-specific membrane antigen (PSMA) PET/CT is offered to assess the presence of local nodal or extensive metastatic disease. Oligometastatic disease, which is PCa with a limited number of 1 to 5 metastatic deposits, possibly provides a time window to delay the course of the disease, or perhaps even still cure the patient. To detect these first metastases in small or normal-sized lymph nodes, the most sensitive, noninvasive imaging method should be selected. In this review, we summarize the current use of MRI and PSMA-PET/CT and discuss the latest developments in these techniques that could further improve initial diagnosis and staging of oligometastatic PCa.

Prostate cancer (PCa) is the most commonly diagnosed cancer among men in 118 of the 185 countries, including many European nations. Its incidence varies markedly across different regions, with higher incidence rates (35.5 per 100,000 men) in transitioned countries compared with transitioning countries (12.6 per 100,000 men).^1^ Access to early detection and effective treatment in high-income countries explains the disparity between incidence and mortality rates, of which the latter are highest in the Caribbean and sub-Saharan Africa.^1,2^ As with many solid malignancies, cancer localized within the prostate is not lethal; it is its extensive metastases from lymph nodes (LNs) to bones, liver, and lungs that are the final cause of death. The European Association of Urology (EAU) classifies PCa into risk groups—low, intermediate (favorable and unfavorable), and high—based on prostate-specific antigen (PSA) levels, Gleason score, and clinical T-stage.^3^ This classification helps guide treatment decisions by assessing the likelihood of cancer progression and spread. Identifying the correct risk group is crucial for selecting the most appropriate additional imaging techniques and treatment strategy. The early stage of metastatic involvement, with a limited number of 1 to 5 metastatic deposits, is called oligometastatic disease,^4–6^ Improving the assessment of oligometastatic disease, by detecting these first metastases in small or normal-sized LNs, can provide a window of opportunity to treat patients with curative intent. In this review, we describe the current and future role of advanced imaging in the initial diagnosis and staging of PCa, with a focus on oligometastatic disease.

## TRADITIONAL STAGING OF LYMPH NODE METASTASES

For many years, the presence or absence of nodal disease involvement has been used to determine whether treatment would aim for cure or palliation, making accurate lymph node staging (N-staging) crucial for therapy planning.^7^ Traditionally, this involved a surgical (extended) pelvic lymph node dissection (ePLND) with histopathologic evaluation of resected nodes.^8^ This procedure is invasive and carries a considerable complication rate.^9^ Moreover, it may miss nodes outside the dissection field (eg, pararectal, internal iliac areas)^10–12^ and small nodes within the area, leading to underestimation of metastatic load or even false-negative results.^10^ In addition, removing healthy nodes offers no benefit and increases complication risks.

Evidence for a direct therapeutic benefit of an ePLND has not been supported by recent studies,^13^ and EAU guideline updates of 2024 have removed the strong recommendation for ePLND in all high-risk PCa patients, emphasizing that its primary role is diagnostic rather than of proven therapeutic benefit. A large study argued against the use of a salvage PLND as metastasis-directed therapy alone for patients with node-only recurrent PCa. These men should instead be considered at high risk of systemic dissemination, already at the time of nodal recurrence,^14^ and be treated as such, as proposed earlier by the same group.^15^

## MULTIPARAMETRIC MRI OF THE PROSTATE

After multiple high-level evidence diagnostic studies,^16–18^ the initial diagnosis of PCa has undergone a major shift with the introduction of a multiparametric MRI (mpMRI) examination.^19^ mpMRI is now recommended as the first-line diagnostic modality before biopsy^3^ in men with elevated PSA levels and/or an abnormal digital rectal examination, enabling targeted biopsies of tumor-suspicious areas. A clinical mpMRI examination includes T2-weighted (T2w), diffusion-weighted (DWI), and gadolinium-based dynamic contrast-enhanced MR images (DCE).^20^ Both the acquisition of these sequences and their interpretation by radiologists have been standardized in the most recent Prostate Imaging Reporting and Data System (PI-RADS v2.1).^21^ Once cancer is confirmed in either MR-guided or systematic multicore needle biopsies, the local radiologic tumor stage, in combination with its histopathologic grade group and PSA level, determines the risk group and the likelihood of developing metastatic disease. For this likelihood, identification of extracapsular extension of the primary tumor on mpMRI as the local radiologic tumor stage is an important determinant.

## WHOLE-BODY (DIFFUSION-WEIGHTED) MRI FOR STAGING METASTATIC DISEASE

The use of MRI to detect or monitor bone metastases has its roots in the early 2000s, but its more widespread use and the demand for a more comprehensive whole-body MRI (WB-MRI) approach to assess node and bone metastases appeared later.^22,23^ Originally, WB-MRI relied on basic T1w and T2w pulse sequences with fat suppression by inversion recovery, which provided essential contrast for bone marrow and soft-tissue evaluation but had complicated workflows requiring patient repositioning and breath-holding. Nowadays, WB-MRI employs optimized sequences such as the T1w gradient recalled echo with Dixon technique enabling fat-water separation through in-phase, opposed-phase, water-only, and fat-only reconstructions, a T2w turbo spin echo sequence, and DWI providing functional insights into tissue cellularity.^24^ With whole-body surface coils, the use of 3T magnets, parallel imaging, and rolling bed platforms, scan times can be kept to under 1 hour. In consensus among some of the most prominent developers and users of the technique, the MET-RADS-P system was proposed: the METastasis Reporting and Data System for Prostate Cancer.^25^ MET-RADS-P generally defines lesions ≥1.5 cm in the longest diameter as the lower threshold of reliably detectable lesions, due to the resolution of especially DWI. Although in some cases this threshold can be lowered to ∼1 cm, if spatial resolution allows, it still means that normal-sized LNs cannot be adequately assessed for the presence of metastases with this technique. If DWI acquisitions are accelerated further, or when focused on, for example, only the lower abdomen, the attainable spatial resolution of DWI (∼2×2x3 mm) is high enough to detect LNs smaller than 1 cm, but distinguishing between benign and malignant nodes remains very difficult, as both express high restrictions for water motion due to the presence of many cells and cellular structures.

## NANOPARTICLE-ENHANCED MRI FOR STAGING NODAL METASTASES

This size limitation leaves room for another imaging method that can reliably identify metastatic involvement in LNs, especially in those of small and normal size. MRI enhanced with the aid of ultrasmall superparamagnetic iron oxide (USPIO) nanoparticles is such a method. Ferumoxytol and ferumoxtran-10 are 2 USPIO nanoparticles that have been around in (pre-)clinical research for over 25 years now (under the brand names Feraheme for ferumoxytol and Sinerem, Combidex, and currently Ferrotran for ferumoxtran-10).^26^ Ferumoxytol is FDA-approved and available in the USA as an iron replacement product to treat iron deficiency anemia in patients with chronic kidney disease, and can be used off-label for USPIO-enhanced MRI. Ferumoxtran-10 is more lymphotropic, and its intended use is the visualization of lymph node metastases with USPIO-enhanced MRI. Ferumoxtran-10 is not FDA-approved, but is available for clinical studies and on terms of Named Patient Use Programs in the Netherlands and in Switzerland, and a phase III international (the Netherlands, Switzerland, and Germany) multicenter pivotal trial aimed for Marketing Authorization Approval has just reached its primary and secondary endpoints. Ferumoxtran-10 was designed for prolonged presence in circulation (blood half-life of 24 to 36 h^27^), intended to be phagocytosed by macrophages, accumulating in healthy LNs. This USPIO accumulation in LNs disrupts local magnetic field homogeneity, causing attenuation or loss of MR signal on T2*w imaging. Contrarily, in metastatic (parts of) LNs, macrophages do not (or less so) phagocytize the nanoparticles, rendering these (parts of) nodes to retain their MR signal intensity. With a blood half-life of 10 to 14 hours, ferumoxytol accumulates less efficiently in LNs, requiring a 3-fold higher dose^28^ (7.5 vs. 2.6 mg Fe/kg for ferumoxtran-10) to attenuate the MR signal, still with residual overlap in signal intensity between metastatic and nonmetastatic nodes.^29^ A meta-analysis on USPIO-enhanced MRI with ferumoxtran-10 reported sensitivities of up to 90% and specificities of up to 96% for detecting nodal involvement across various cancers.^30^ Of note, data from these studies are over 15 years old now, biased towards currently outdated MRI acquisition schemes, favoring larger LNs (due to 2D multislice approaches instead of current isotropic high-resolution 3D pulse sequences). In a more recent single-institution study with ferumoxytol in 39 patients, sensitivity and specificity for metastatic LN detection were 98% and 64%.^29^

## TECHNICAL DEVELOPMENTS IN MPMRI DATA ACQUISITION OF THE PROSTATE

PI-RADS^31^ provides guidance for the standardization of data acquisition protocols for mpMRI of the prostate.^21^ Recent advancements within these guidelines focus on reducing scan times without changing contrast or spatial resolution of pulse sequences, hence maintaining high diagnostic quality. The introduction of deep learning reconstruction has significantly reduced acquisition times by 65%,^32^ whereas advanced compressed sensing reduced scan times by 26%^33^ for T2w prostate images. All major MR system manufacturers nowadays have incorporated some sort of AI-enhanced acquisition, denoising, and reconstruction in their portfolios. Stepping away from PI-RADS guidance, numerous studies have demonstrated potential improvements in the prostate acquisition framework. Magnetic resonance fingerprinting (MRF) enables a fast and simultaneous generation of multiple quantitative property maps (T1, T2) of the prostate that are inherently spatially registered and motion robust.^34^ Along with traditional ADC maps, it can distinguish between cancerous and normal prostate tissue.^35^ A 3-dimensional prostate MRF acquisition produces high-resolution quantitative T1 and T2 images within 4 minutes,^36^ instead of T1 and T2 weighted scans of 3.5 minutes each. The added value of this and other T1 quantification methods, based on Look-Locker inversion recovery^37^ or single-shot T1FLASH,^38^ for the accuracy of PCa detection and characterization, is still unknown.

Diffusion-weighted imaging of the prostate is also subject to all kinds of improvements. PI-RADS guides the choice of *b*-values, and the way to extrapolate monoexponential fits to the signal decay curve toward the calculation of high *b*-values has been known for many years.^39^ Modeling signal decay from multiple diffusion directions and multiple *b*-values^40^ has been reviewed for studies interrogating prostate (cancer) tissue microstructure, so far with comparable diagnostic accuracy.^41^ Recently, even the diffusivity of metabolites in the prostate has been assessed for this purpose, separating luminal and intracellular compartments on the basis of metabolite motion.^42^

Mainstream prostate mpMRI is performed at magnetic field strengths of 1.5 and 3 T, but feasibility at lower (0.55 T)^43^ and higher (7 T)^44,45^ field strengths has been shown as well.

For now, these additional or quantitative acquisition protocols extend the duration of standard mpMRI examinations, whereas the clinical trend—driven by high demand and limited MRI scanner availability—is to reduce clinical examination times. Since radiologists primarily rely on T2w MRI and DWI for PI-RADS assessment, one popular approach is to omit contrast agent administration in a so-called biparametric or noncontrast prostate MRI exam, especially for primary detection or screening. This approach reduces preparation time (no IV injection required), removes contrast agent costs, shortens examination time, and has been shown in a large international multicenter observer study to be noninferior to mpMRI in detecting clinically significant PCa.^46^ The major caveats of biparametric MRI are the need to acquire high-quality data and the requirement for highly experienced readers. If motion artifacts degrade anatomic resolution in T2w MRI, or if rectal gas or hip implants compromise DWI, the absence of DCE significantly limits diagnostic confidence. The Prostate Imaging Quality system PI-QUALv2,^47^ developed to systematically evaluate the quality of bi- or mpMRI examinations, can help determine whether repeat imaging is warranted in patients with inadequate scan quality.

## PSMA-PET/CT FOR STAGING NODAL METASTATIC DISEASE

Contrary to the mere use of nodal size and anatomic appearance on MRI or CT as a noninvasive indicator of nodal metastases, prostate-specific membrane antigen (PSMA) PET/CT imaging is functionally targeted toward PCa cells in LNs and has emerged as a valuable tool for detecting metastatic nodes over the last decade. PSMA is a protein highly expressed on the surface of PCa cells, with only 5% of primary prostate tumors being PSMA-negative. Small-molecule PSMA ligands bind to the active site within the extracellular domain of PSMA, are internalized and endosomally recycled by cancer cells, leading to enhanced tumor uptake and retention.

From the introduction of [^68^Ga]Ga-PSMA-11 in 2011, different ^68^Ga- or ^18^F-radiolabeled PSMA radioligands have been developed and used in clinical practice. While the ease of isotope production using a germanium-gallium generator and radiolabeling of the tracer are pros for using ^68^Ga-PSMA tracers, ^18^F-labeled compounds have been adopted for their longer half-life, later time point imaging capacity, and large-scale, centralized production. Regarding the ^18^F-radiolabeled PSMA tracers, [^18^F]DCFPyL is largely used for its stable cyclotron production and improved tumor-to-background ratio, with less non-specific bone activity often seen in [^18^F]PSMA-1007. Other tracers like [^18^F]rhPSMA-7.3 are also increasingly used, based on encouraging results from phase 3 trials [ie, LIGHTHOUSE (NCT04186819) and SPOTLIGHT (NCT04186845)].

This imaging technique outperforms anatomic imaging by CT or MRI with good specificity (67% to 100%, pooled 0.95), though high variation in reported sensitivity (23% to 100%, pooled 0.58) exists.^48,49^ Sensitivity is hindered by the spatial resolution of PET/CT, around 4 to 5 mm, which still makes it difficult to detect LNs smaller than 5 mm. In addition, this imaging method may fail to detect LNs that express little or no PSMA. There is also the challenge of distinguishing between physiological, benign, and PCa-related pathologic uptake. Next to well-known areas of physiological PSMA expression (salivary glands, liver, spleen, proximal small bowel, and urinary bladder), mild to moderate PSMA expression has been reported in “pitfalls,” for example, the celiac, presacral, and stellate ganglia,^50,51^ as well as in pleural plaques and Pagetoid bone.^52^ This complicates accurate diagnosis and could require an additional learning curve for the observers. Moreover, appropriate clinical guidelines should be developed for the harmonization of the interpretation of PSMA-PET results. Diverse practice guidelines have been proposed^53,54^ as educational tools designed to assist practitioners in providing appropriate care for patients, with PRIMARY and PROMISE criteria showing potential to increase the diagnosis of csPCa and increase the accuracy of interpretation of scans.^55^

## DEVELOPMENTS IN HYBRID PSMA-PET/CT AND PET/MRI

As a hybrid imaging modality that combines morphologic, functional, and molecular information, PSMA-PET/CT enhances diagnostic performance for TNM staging in patients with PCa. The introduction of hybrid [^18^F]FDG-PET/MRI imaging also offers encouraging results in diagnosing PCa, with added value in both localized disease and in the oligometastatic setting. MRI outperforms CT in soft-tissue contrast, improved bone marrow evaluation, and the absence of radiation exposure.^56^ Nowadays, long axial field of view PET-CT scanners are increasingly used in clinical practice. Their main advantage is the improvement in lesion detectability of small (oligo-)metastatic lesions. This allows a reduction of the injected radiotracers dose, while reducing the scan time with increased diagnostic accuracy. The marked reduction of radiation exposure is favorable in an oncological setting, where patients undergo longitudinal scans for diverse purposes, often including response evaluation.^57^ Irrespectively of the type of hybrid scanner used, the combined approach of dual tracers applied (eg, [^18^F]FDG and radiolabeled-PSMA) may also assist in improving primary tumor detection and revealing the metabolic heterogeneity of PCa.

When comparing the role of ^18^F-PSMA-1007 PET/CT and mpMRI for local staging in patients with intermediate and high-risk PCa, using histopathology as the gold standard, dual imaging was found to improve T-staging of primary PCa.^58^ However, the addition of PSMA-PET/CT to mpMRI was found to be only borderline cost-effective by reducing futile biopsies in, and the diagnosis of, low-risk PCa. In men with negative mpMRI, the role of PSMA-PET/CT was not cost-effective due to minimal improvement in detecting clinically significant PCa, while increasing the number of unnecessary biopsies.^59^ Recently, the discrepancy between mpMRI and PSMA-PET/CT of the prostate was retrospectively evaluated in 309 patients with clinically significant PCa who underwent both imaging modalities.^60^ Whereas major discordances were reported in 39 patients (N=39), on patient level only 6 patients had an mpMRI PI-RADS score of 1 or 2, and only 13 had PI-RADS 3, questioning again whether adding PSMA-PET.CT to an mpMRI examination for local disease detection would be cost-effective.

In a head-to-head comparison for the detection of lymph node metastases, the sensitivity and specificity of PSMA-PET/CT (73.7% and 97.5%) were found to be superior to conventional anatomic CT (38.5% and 83.6%) and mpMRI (38.9% and 82.6%).^61^ This higher diagnostic accuracy was primarily due to PSMA-PET/CT not depending on a size criterion, enabling the detection of normal-sized metastatic nodes. Small-molecule PSMA ligand imaging detects active metastases before structural bone changes on CT or MRI. Therefore, PSMA-PET/CT is a suitable replacement for conventional anatomic imaging, based on a 27% higher accuracy in identifying pelvic LN or distant metastases.^62^ Still, there is a lower limit in the size of detectable metastases in LNs with current PSMA-PET/CT systems. In extensive comparisons with histopathology of removed LNs after PLND, PSMA-PET/CT identified approximately one-third of the positive nodes, with the median size of LNs missed of 3 to 5 mm. This indicates a size-dependent sensitivity for PSMA-PET/CT.^63–65^

## FUTURE DIRECTIONS IN IMAGING OLIGOMETASTATIC DISEASE

As with many different types of solid cancers, PCa has a high propensity to metastasize first to locoregional and distant LNs. The early stage of metastatic involvement, with a limited number of 1 to 5 metastatic deposits, so-called oligometastatic disease,^4–6^ possibly provides a time window to delay the course of the disease, or perhaps even still cure the patient. To detect these first metastases in small or normal-sized LNs, the most sensitive, noninvasive imaging method should be selected. This is necessary, as the mean size of LNs in the pelvis, as measured with MRI at an ultrahigh field strength in healthy volunteers, is only 2 to 3 mm in short axis.^66^ The number of pelvic LNs varies per person, as was shown in superextended PLNDs of 30 cadavers, harvesting between 24 and 60 pelvic LNs,^67^ quite in line with the number of nodes detected with the MRI study at 7 T, ranging from 19 to 90.^66^ As previously mentioned, 3 relatively recent competitive or complementary imaging techniques are available for the detection of metastatic LNs of PCa: PSMA-PET/CT, WB-MRI, and USPIO-enhanced MRI.

**FIGURE 1:**
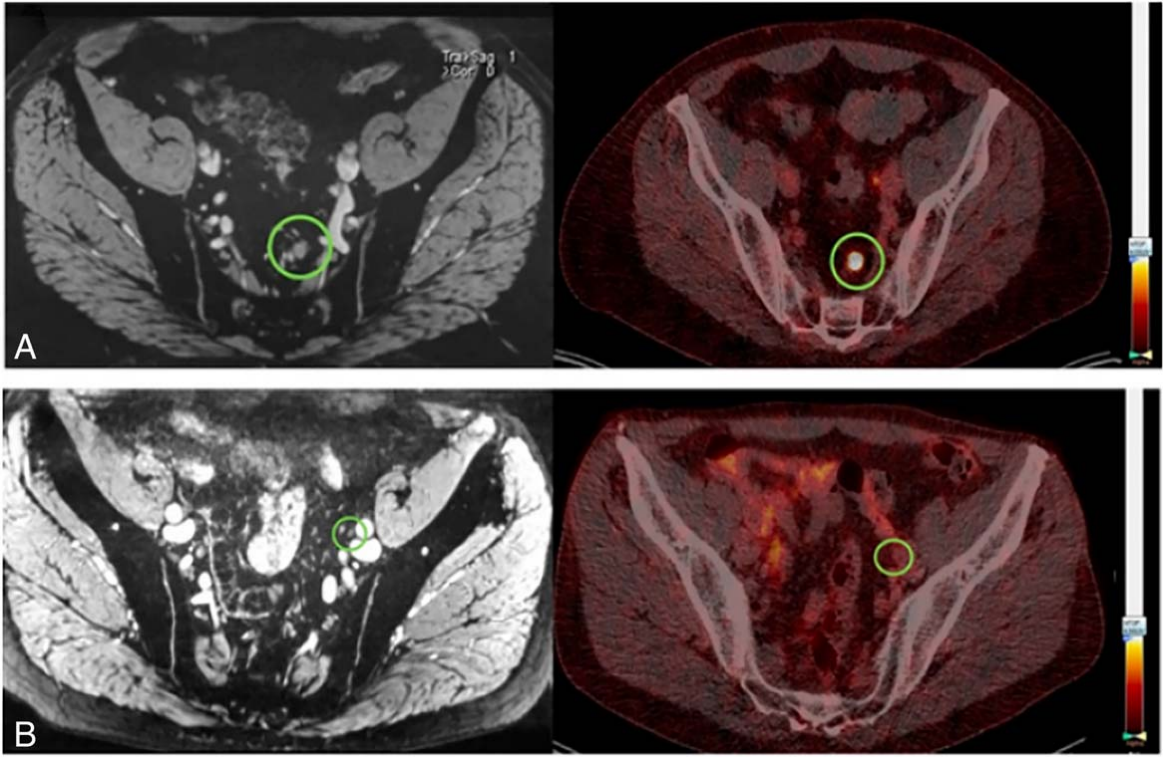
MRI and PSMA-PET/CT images from a patient with metastatic PCa. Iron-sensitive T2*w fat-saturated USPIO-enhanced MRI (left) and the corresponding fused PSMA-PET/CT scans both identify a 7-mm lymph node in the left pararectal region (green circle in A). A 4-mm lymph node adjacent to the left external iliac artery is positive on MRI only, but remains undetected on PSMA-PET/CT (green circle in B). Partially reproduced with permission from Schilham et al.^68^

**FIGURE 2:**
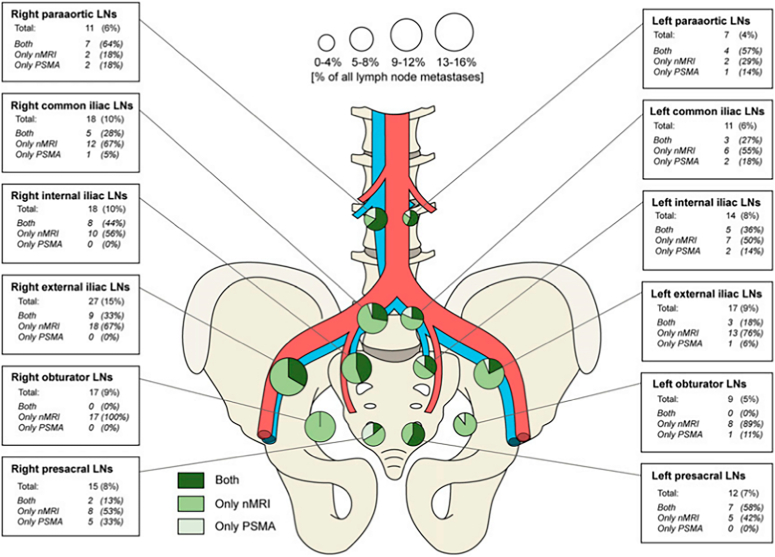
Anatomic distribution of identified suspicious LNs as detected by USPIO-enhanced MRI (nMRI) and PSMA-PET/CT in 45 patients with PCa. The circle size reflects the percentage of suspicious nodes, and the green colors identify the imaging modalities. Reproduced with permission from Schilham et al.^68^

**FIGURE 3:**
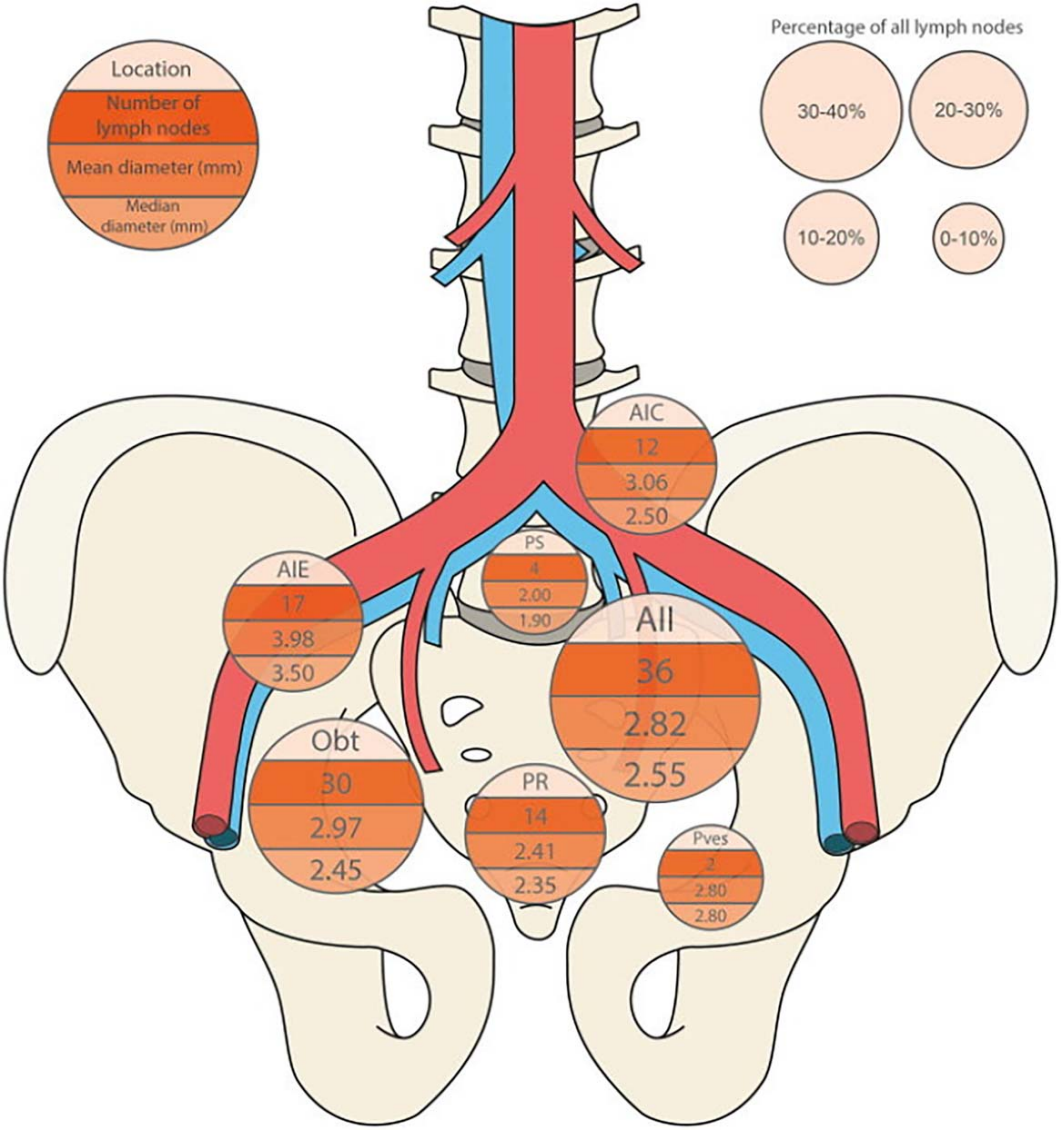
A summary of location, size, and average number of MRI-positive LNs, detected in 20 patients examined at an ultrahigh magnetic field strength of 7 Tesla with USPIO-enhanced T2*w MRI. All patients had PCa with a high risk of nodal disease. Note the small median diameters of these nodes, below 3 mm in all but one anatomic location. AIC indicates common iliac artery; AIE, external iliac artery; AII, internal iliac artery; LN, lymph node; LoS, level of suspicion; MRI, magnetic resonance imaging; Obt, obturator region; PR, pararectal; PS, presacral; Pves, perivesical; USPIO, ultrasmall superparamagnetic iron oxide. Reproduced with permission from Fortuin et al.^71^

When trying to detect the earliest phase of nodal involvement, which of these imaging techniques should we use? The MET-RADS-P system identified a lower detection threshold of lesions of 1.5 cm long axis for WB-MRI,^25^ which rules out this method for detecting metastases in small or normal-sized nodes. Even if DWI detects normal-sized nodes, discrimination between benign and metastatic is problematic. PSMA-PET/CT and USPIO-enhanced MRI are preferred due to their higher sensitivity. To evaluate the similarities and differences between these 2 modalities in the same patients, the number, size, and anatomic location of suspicious LNs in 45 patients with PCa who underwent both scans were compared (example in Fig. 1), albeit without histopathology of the identified suspicious nodes.^68^ USPIO-MRI identified significantly more suspicious LNs per patient (mean: 3.6) than ^68^Ga-PSMA-PET/CT (mean: 1.6), and the suspicious LNs identified by USPIO-MRI were significantly smaller (Fig. 2). Both imaging modalities have also been compared ex vivo in a separate study of excised suspicious LNs. Removed nodes, after injection of USPIO (2 d before surgery) and ^111^In-PSMA, were examined with SPECT and USPIO-enhanced MRI on an 11.7 T preclinical MR system. Even on the subnodal level, correspondence between the 2 modalities and histopathologic PSMA expression was excellent (90%) or good (7%).^69^ The sensitivity for detecting the smallest suspicious nodes in vivo could even be further increased by using USPIO-enhanced MRI at an ultrahigh magnetic field strength of 7 T. In a direct comparison of 20 patients examined at 3 and 7 T, more and smaller suspicious nodes were detected with higher confidence at the higher field strength, detecting nodes with short axes down to 1.5 mm in size.^70^ Two radiologists annotated the location within the pelvis and size of these suspicious nodes: more than 63% of these nodes were smaller than 3 mm, and many were located outside the surgical field of an ePLND (Fig. 3).^71^ This small size and atypical locations render it sheer impossible to perform an ePLND that would remove all preoperatively identified suspicious LNs. Even if removed surgically, identifying these small nodes in resected tissue to perform node-to-node matching of in vivo imaging with histopathology of the same nodes appears similarly daunting. Without a matching histopathologic reference standard, alternative validation methods need to be developed. In future studies, one could consider using traditional size criteria by longitudinal follow-up on the size of small suspicious LNs in patients with an unexpected rise in PSA after prostatectomy. Another study possibility could be to treat these small suspicious LNs with stereotactic radiotherapy and monitor decreasing PSA levels of these patients as indirect validation of the nodes being metastatic.

Next to detecting the smallest metastatic LNs, increasing certainty of the absence of lymph node metastases is of equal importance. Due to the significant proportion of patients undergoing ePLND without ultimately harboring LN metastases at final pathology,^72,73^ the associated complications of the surgery,^74^ and the absence of strong evidence supporting the oncological benefit,^9^ it is urgent to optimize the indication for ePLND. Current clinical practice often employs considerable overtreatment for precautionary reasons. Knowledge of the absence of nodal invasion in the individual patient would fit in with trends toward safely avoiding an ePLND. However, when oligometastatic disease is identified, there may be an opportunity for targeted radiation therapy, such as MR-guided stereotactic body radiotherapy (SBRT) using a combined MRI and linear accelerator system (MR-LINAC), potentially with curative intent. Even if metastases are later identified elsewhere, further treatment can still be pursued following the initial targeted therapy. Conducted trials suggest that metastasis-directed therapies—particularly SBRT—can prolong androgen deprivation therapy-free survival and delay the initiation of systemic therapy in patients with oligometastatic disease.^75^

## CONCLUSION

Altogether, the clinical research field in diagnostic imaging of PCa is dynamic. We have summarized the different advanced imaging modalities described in this review for the detection of nodal metastases in PCa in Table 1. This table shows the pros and cons of the different techniques with their most appropriate clinical indications. The current preferred mainstream diagnosis of localized disease is mpMRI, in which many new additions are under investigation, but need to be validated in large prospective clinical studies to replace or complement the current mpMRI protocols. PSMA-PET/CT has revolutionized the approach to staging PCa for the presence of metastases. Its diagnostic use has expanded significantly since its introduction. The demonstrated superior accuracy over conventional imaging for staging pelvic LN metastases and distant metastases due to PSMA targeting cancer cells emphasizes an increasing role in guiding therapeutic decisions and improving patient outcomes. When available, USPIO-enhanced MRI appears to be even more sensitive for detecting or excluding small lymph node metastases. The evaluation of high-resolution 3D USPIO-enhanced MRI scans requires considerable radiologic effort, and the exact clinical relevance of detecting the earliest or smallest lymph node metastases still needs to be proven. Until then, PSMA-PET/CT is favored for its relative ease in scan interpretation and the additional benefit of detecting distant metastases, particularly in bone.

**TABLE 1:** Comparative Overview of Advanced Imaging Modalities for the Detection Of Nodal Metastases in Prostate Cancer

Modality	Advantages	Limitations	Most Appropriate Clinical Indications
PSMA-PET/CT	High sensitivity and specificity for nodal and distant metastases Detects lesions not visible on conventional imaging Whole-body coverage in a single examination Increasingly available and incorporated into clinical guidelines	Reduced sensitivity for small (<5 mm) nodal metastases False negatives in tumors with low or heterogeneous PSMA expression Radiation exposure Limited access in some regions	Primary staging in intermediate-risk and high-risk prostate cancer Restaging in biochemical recurrence Identification of oligometastatic disease to guide metastasis-directed therapy Patient selection for PSMA-based Radioligand therapy
Whole-body MRI (WB-MRI)	Radiation-free, repeatable modality Whole-body evaluation of nodes and bone in one session Useful for systemic disease assessment	Lower sensitivity for small nodal deposits compared with PSMA-PET Limited availability and expertise in some centers Longer examination times may reduce patient compliance	Comprehensive staging when radiation exposure should be avoided (eg, younger patients, follow-up) Simultaneous assessment of nodal and osseous disease Potential alternative when PSMA-PET is unavailable
USPIO-enhanced MRI	High sensitivity for small (<5 mm) nodal metastases Provides functional information on nodal microarchitecture Outperforms PSMA-PET and conventional MRI in nodal staging	Contrast agent (iron oxide nanoparticle) not yet EMA approved More complex logistics and longer imaging protocols Restricted mainly to specialized centers and research settings	Selected high-risk patients requiring highly accurate nodal staging Research studies Problem-solving tool when standard imaging is inconclusive

PSMA-PET/CT provides high sensitivity and specificity and is increasingly incorporated into international guidelines, particularly for staging and recurrence assessment. Whole-body MRI offers radiation-free, comprehensive systemic evaluation and standardized reporting frameworks, but remains less sensitive for sub-centimeter nodal deposits compared with PSMA-PET. USPIO-enhanced MRI demonstrates excellent sensitivity for small nodal metastases, although its use is currently largely limited to research settings, awaiting EMA approval.

CT indicates computed tomography; MRI, magnetic resonance imaging; PET, positron emission tomography; PSMA, prostate-specific membrane antigen; USPIO, ultrasmall superparamagnetic iron oxide.
